# Fungal G-Protein-Coupled Receptors: A Promising Mediator of the Impact of Extracellular Signals on Biosynthesis of Ochratoxin A

**DOI:** 10.3389/fmicb.2021.631392

**Published:** 2021-02-12

**Authors:** Jing Gao, Xinge Xu, Kunlun Huang, Zhihong Liang

**Affiliations:** ^1^Beijing Laboratory for Food Quality and Safety, Beijing, China; ^2^College of Food Science and Nutritional Engineering, China Agricultural University, Beijing, China

**Keywords:** G protein-coupled receptors, ochratoxin A, quorum sensing, oxylipin, trans-kingdom, transcription factor

## Abstract

G-protein-coupled receptors (GPCRs) are transmembrane receptors involved in transducing signals from the external environment inside the cell, which enables fungi to coordinate cell transport, metabolism, and growth to promote their survival, reproduction, and virulence. There are 14 classes of GPCRs in fungi involved in sensing various ligands. In this paper, the synthesis of mycotoxins that are GPCR-mediated is discussed with respect to ligands, environmental stimuli, and intra-/interspecific communication. Despite their apparent importance in fungal biology, very little is known about the role of ochratoxin A (OTA) biosynthesis by *Aspergillus ochraceus* and the ligands that are involved. Fortunately, increasing evidence shows that the GPCR that involves the AF/ST (sterigmatocystin) pathway in fungi belongs to the same genus. Therefore, we speculate that GPCRs play an important role in a variety of environmental signals and downstream pathways in OTA biosynthesis. The verification of this inference will result in a more controllable GPCR target for control of fungal contamination in the future.

## Introduction

Ochratoxin A (OTA), which is a type of mycotoxin produced mainly by various *Aspergillus* and *Penicillium* species (Frisvad et al., 2019), was classified as a group IIB carcinogen by the International Agency for Research on Cancer (IARC) (International Agency For Research On Cancer, 1993). The latest *in vitro* toxicology experiment showed that alteration of DNA methylation occurred? in OTA-induced G0/G1 phase arrest (Zhang et al., 2019). Numerous cell and animal experiments have reported that OTA exposure can cause many toxicological effects, which include teratogenicity, carcinogenicity, mutagenicity, hepatotoxicity, and was a main causative agent of human Balkan endemic nephropathy (Pfohl-Leszkowicz and Manderville, 2012; Zheng et al., 2013; Qi et al., 2014; Zhang et al., 2014; Zhao et al., 2017; Hou et al., 2020). OTA widely contaminates feed and food commodities, and it is accumulated through the food chain in animals (Wenying et al., 2018). Global warming promotes the poleward movement of toxigenic fungi, causes contamination in previously unsuitable geographic regions, and exacerbates this threat (Bebber et al., 2013). OTA pollution occurs at various stages of agricultural production (i.e., plant growth, harvest, processing, transportation, and storage), and the occurrence of toxigenic fungi and the biosynthesis of OTA was influenced significantly at different stages of synthesis by external environmental factors, signaling molecules, and trans-kingdom communication.

Sensing and responding to environmental fluctuations are crucial to the survival of microorganisms. GPCRs are ubiquitous and the largest family of transmembrane receptors in both prokaryotes and eukaryotes. They contain seven transmembrane domains (TMDs) and are connected by alternating intracellular coils (IC1-IC3) and extracellular loops (EC1-EC3) (Baldwin, 1993). The extracellular amino acid? fragments sense the environment by interacting with a diverse array of ligands, then transmit this interaction through protein folding modifications to the intracellular carboxy-terminal end that recognizes the cognate heterotrimeric G proteins (Gαβγ) (Ballesteros et al., 2001; Shapiro et al., 2002). Sensitization of a GPCR by ligands results in the exchange of GTP for GDP on the Gα subunit, which leads to the dissociation of Gβγ; both GTP-Gα and Gβγ dimers can interact with respective effectors to activate or inhibit specific downstream pathways (Khan et al., 2013; Brown et al., 2018). In fungi, GPCR-regulated signaling pathways that include the cAMP-activated Protein Kinase A (PKA) pathway (Xue et al., 2006), mitogen-activated protein kinases (MAPK) cascades pathway (Chen and Thorner, 2007; Atoui et al., 2008; Hamel et al., 2012; Ma and Li, 2013), and the phospholipase C (PLC) pathway (Ansari et al., 1999) influence gene expression to regulate cell growth, morphogenesis, mating, stress responses, and metabolism in a complex and intersecting way (Rispail et al., 2009).

**FIGURE 1:**
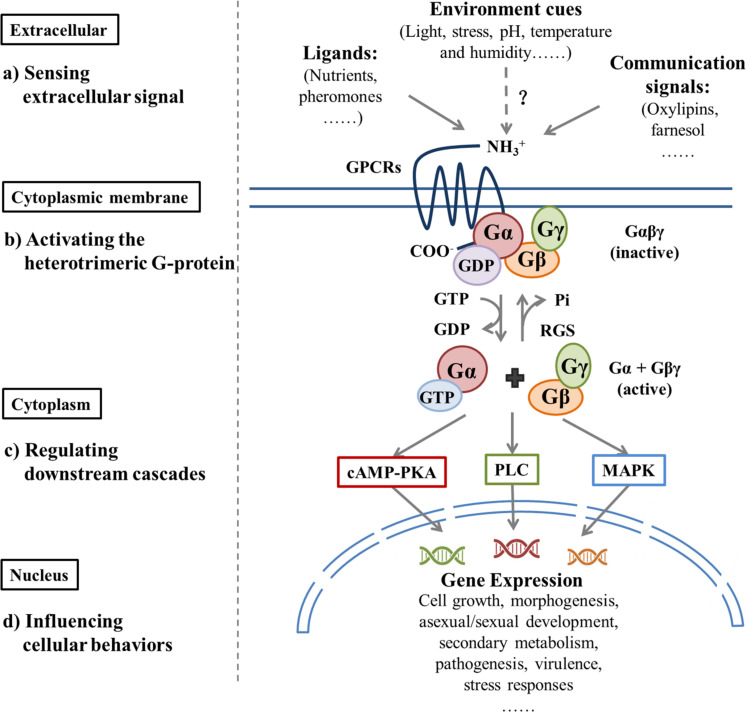
Extracellular signals regulate downstream pathways by activating G protein signaling and, thus, influence cellular behavior.| Different classes of GPCRs sense various extracellular factors, which include ligands, environmental cues, and communication signals that bind to the GPCR; this causes GDP-GTP exchange on the Gα protein and dissociation of Gα and Gβγ. Both the Gα-GTP and Gβγ dimers may trigger respective downstream signal cascades, which include the cAMP-activated Protein Kinase A (PKA) pathway, mitogen-activated protein kinase (MAPK) cascades pathway, and the phospholipase C (PLC) pathway. This process influences cellular growth, reproduction, metabolism, virulence, and stress responses. GTP hydrolysis by the Gα subunit results in the reassociation of Gα-GDP with the Gβγ dimer and GPCR, which completes the G protein cycle. The Regulator of G-protein Signaling (RGS) proteins can inactivate G protein signaling rapidly by increasing intrinsic GTPase activity of GTP-bound Gα subunits. In this figure, dashed arrows with the question mark (?) indicates hypothetical interactions.

The regulator of G protein signaling (RGS) is a GTPase activating protein (GAP), which accelerates the decomposition of GTP-binging Gα by enhancing Gα subunit GTPase activity that negatively regulates G protein signal transduction (Kasahara et al., 2000; McPherson et al., 2018). Another G protein signal-regulating protein, Phosducin-like protein (PhLP), is involved in the G protein signal transduction pathway by positively regulating Gβγ subunits (Lukov et al., 2014). Significant differences exist in the abundance and diversity of these receptors in fungi and the potential ligands that they detect. A variety of GPCRs relates to OTA biosynthesis, which involves a wide range of environmental signals and downstream pathways (Figure 1). Because of their cell surface location and central mediating role, GPCRs are specific targets to control the biosynthesis of fungal toxins and to intervene in fungal disease and mycotoxin contamination.

The well-researched GPCR-mediated, G-protein signaling pathway, which regulates fungal behavior in response to a variety of signals, is highly conserved in animals, plants, and microorganisms. This phenomenon and the mechanism of model strains have been studied thoroughly enough to provide the guiding ideology and practical basis for research on GPCRs in fungi. The bovine rhodopsin and human beta2-adrenergic GPCRs were crystallized and based on homology and structural similarity, which served as models for the structure of other members of the GPCR family (Stenkamp et al., 2002; Rasmussen et al., 2007). Although GPCR signaling is essential for fungal biology, the identified interactions of GPCR–G-protein, the studies of receptor binding ligands, and the resolved GPCR crystal structures are only the beginning of what we need to know. Perfecting the above information will prove vital in understanding the upstream ligand–receptor relationship of the OTA synthesis regulatory pathways and the development of novel fungal GPCR-targeting of mycotoxin control. This paper reviews the current understanding of fungal GPCR-mediated signaling pathways that regulate fungal behavior and OTA synthesis; this has important theoretical significance for improving our knowledge about the function of GPCRs and the synthesis of secondary metabolites. It is expected that we will eventually use GPCR as targets to prevent and control the harm caused by fungal toxins.

## GPCRs Sense Nutrients and Pheromones

Fungal adaptation to distinct microenvironments, which include hosts, natural habitats, and culture media, is essential for their success (Brown and Goldman, 2016). G-protein signaling pathways play a vital role in sensing external ligands, which include nutrients, hormones, proteins, and peptides (including pheromones), ions, hydrophobic surfaces, and light (Kochman, 2014). This enable fungi to coordinate function, metabolism, and development that, in turn, promotes their survival, propagation, and virulence (Van Dijck et al., 2017). At present, based on structural similarity and homology, fungal GPCRs are classified into 14 categories, which include six original classes and eight novel classes (Supplementary Table 1; Lafon et al., 2006; Li et al., 2007; Zheng et al., 2010; Grice et al., 2013; Cabrera et al., 2015; Brown et al., 2018; Nadarajah et al., 2018). The model filamentous ascomycete fungus *A. nidulans* possesses 86 putative GPCRs, which are classified into 16 receptors, named GprA∼GprP and NopA, constitute nine categories of GPCRs; only a few have been characterized functionally (Lafon et al., 2006; Krishnan et al., 2012; Brown et al., 2015).

Many studies have found that GPCR-mediated perception of signaling molecules, especially pheromones, nutritions and oxylipins, are closely related to fungal reproduction and the production of secondary metabolites, which seems to imply that there are cross-effects of different GPCRs. This complex regulatory network provides numerous targets for controlling OTA biosynthesis.

### Perception of Nutrients

G-protein-coupled receptors sense nutrients, and then they influence fungal development and metabolism. An enormous amount of basic insights into nutrient utilization by fungi has been established. The interaction of GPCR protein Gpr1 with Gα protein Gpa2 is necessary for the stimulation of cAMP synthesis by sugars. In the model organism *Saccharomyces cerevisiae*, Gpr1 is a high-affinity sucrose and a low-affinity glucose sensor. The addition of glucose to a glucose starvation culture triggers a rapid and transient increase in the cAMP level, which sets off a PKA-mediated protein phosphorylation cascade. Deletion of Gpr1 and/or Gpa2 affected a variety of physiological events controlled by cAMP-dependent protein kinases (cAPKs), such as mobilization of trehalose and glycogen and a rapid loss of stress resistance (Rolland et al., 2000; Lemaire et al., 2004). The absence of Gpr1 can be rescued by the constitutively activated Gpa2^*Val–*132^ allele, which supports the interaction of GPCR and its cognate G protein in regulation (Kraakman et al., 2010). GprC and GprD (Class III) in *Aspergilli* are analogous to Gpr1 in *S. cerevisiae*, which is involved in the sensing of sugar and oxylipin (De et al., 2013). Deletion of the *gprC* or *gprD* gene in *A. fumigatus* affected the ability of strains to produce several toxic secondary metabolites and then affected the growth and pathogenicity in model murine infections (Gehrke et al., 2010). In *A. flavus*, deletion of genes *gprC* and/or *gprD* resulted in alteration of quorum sensing (QS), sporulation, sclerotia formation, and biosynthesis of aflatoxin (AF) (Affeldt et al., 2012). GprK (ClassVI) in *A. fumigatus* was involved in sensing external carbon sources, which is a more complex sensor protein that contains a RGS domain in addition to the 7-TM domains. Overexpression of *gprK* significantly activated toxin in the biosynthesis-related cAMP-PKA pathway, then activated the MAPK signaling pathway, which partially regulates the expression of genes that code for catalase and superoxide dismutase and, thus, indirectly affects secondary metabolism.

Even in the absence of carbon sources, Δ*gprK* mutants showed a severe morphological alteration, a change in asexual reproduction, and a reduction in tolerance to different stressing factors, which included oxidative stress and destruction of the ability to produce gliotoxin (Jung et al., 2016). Both GprM (ClassVII, homologous to rat growth hormone-releasing factor receptors) and GprJ (Class IV, nitrogen source sensors) were involved in regulating the production of DHN-melanin in *A. fumigatus*, and this regulation partially occurred through the activation of MpkA (Manfiolli et al., 2019; Filho et al., 2020). GprH (ClassV), a common glucose and tryptophan sensor, has been found in many filamentous fungi, which regulates hyphal growth and sexual reproduction during carbon starvation in *A. nidulans*. In cultures supplemented with glucose and tryptophan, the deletion of *gprH* caused a decrease in cAMP-PKA activity under stress conditions (Brown et al., 2015), which suggested that a single GPCR interacted with multiple ligands. In *Cryptococcus neoforme*, Gpr4 was similar to Gpr1 in *S. cerevisiae* and to GprH in *A. nidulans*, which induced the cAMP–PKA pathway. Glucose, but not methionine, acted on Δ*gpr4* mutants to cause a change in the cAMP level, and the deficiencies in capsule formation and mating defects were reverted by cAMP supplementation. Therefore, Gpr4 protein has been proposed to be a sensor of amino acids, particularly for methionine (Xue et al., 2006).

There are also examples of other types of nutrient sources that are sensed and regulated by fungal GPCR. Four cAMP-type GPCR genes (*gpr1* to *gpr4*) in *Trichoderma atroviride* belong to class V, and expression of all four *gpr* genes increased after carbon starvation. Gpr1 protein is essential to conidium germination and mycelial growth. Expression of *gpr3* and *gpr4* responded to exogenous cAMP, and the addition of hyphal fragments and cellulosic material also increased the expression of *gpr4* (Brunner et al., 2008). The Pth11 type GPCR of *Neurospora crassa* was involved in the detection of cellulose materials, plant cell walls, or their degradation products, which triggered a response that favored fungal infection of plant hosts (Cabrera et al., 2015; Thieme et al., 2017).

The above results showed that fungi can sense a variety of nutrients through GPCRs, and they can regulate cell growth, development, immune evasion, invasiveness, mycotoxin production, chemotropism, and virulence. Disrupted nutrient-sensing GPCRs could be targeted to reduce fungal virulence and mycotoxin contamination.

### Perception of Pheromones

Pheromones are another type of typical GPCR ligand that influences fungal reproduction and virulence. The benefits of rapid evolution that is driven by sexual reproduction of virulence and resistance are essential for promoting genetic diversity and evolutionary competition with hosts. Pheromones secreted by one type of mating cell and recognized by the opposite type of mating cell trigger downstream signal transduction cascades that lead to cell mating, and they also play a potential role in the production of secondary metabolites (Karlson and Lüscher, 1959; Herskowitz, 1995; Xue et al., 2008; Kim and Borkovich, 2010). In *S. cerevisiae*, the GPCR sex pheromone sensor proteins Ste2 and Ste3 detect the a and α sex peptide pheromones, respectively, which can activate the MAPK cascade to result in cell cycle arrest and cell fusion with the opposite mating type (Burkholder and Hartwell, 1985; Kuchler et al., 1989; Herskowitz, 1995; Chen and Thorner, 2007). PcPRE1 and PcPRE2, which are homologs of Ste2 and Ste3 pheromone receptors involved in mating, have been found in *P. notatum*, *P. chrysogenum*, and *Acremonium chrysogenum;* previously, it was thought that these fungi were incapable of sexual reproduction (Paoletti et al., 2005; Poggeler et al., 2008; Bohm et al., 2013; Terfehr et al., 2014).

The mating-type loci *MAT1-1-1* and *MAT1-1-2* were found in *A. chrysogenum*, and the mating-type genes of the recombinant strains control conidia formation, hyphal differentiation, and penicillin production. In the Δ*mat1* mutant, the expression of biosynthetic genes in penicillin decreased because the pellet size and structure were affected by hyphal differentiation (Poggeler et al., 2008). In *A. nidulans*, GprA and GprB are required for sexual development (without vegetative growth), which include formation of self-fertilized fruiting bodies, asexual development, Hul̈le cell production, and heterothallic sexual development (Han et al., 2004). Single Δ*gprA* and Δ*gprB* mutants and double Δ*gprA*Δ*gprB* mutants were defective in sexual reproduction. Interestingly, GprD-mediated carbon sensing also acted as a repressor of sexual development through the PKA pathway; when grown in the presence of glucose, GprD promoted hyphal growth and conidial germination. The sexual developmental activator NsdD functioned downstream of GprA or GprB, whereas GprD-mediated attenuation of sexual development functioned upstream of GprA and GprB (Han et al., 2004; Seo et al., 2004). On the other hand, GprB and GprD affected QS, spore and sclerotia formation, mycotoxin production, and other metabolic pathways (De et al., 2013). The putative carbon and amino acid receptor GprH worked upstream of the cAMP-PKA pathway, which promoted glucose uptake and hyphal growth and repressed sexual development during carbon starvation.

### Cross-Talk and Interaction Among GPCR-Mediated Pathways

Ligands, GPCRs, and downstream regulatory pathways are not strictly corresponding; this cross-over internal relationship increases the possibility of GPCR as targets for regulating cell behavior. Nutritional state and the perception of sexual partners regulate sexual developmentbecause nutrient limitation reduces pheromone signaling and, in turn, mating efficiency. Nutrient and pheromone GPCR pathways significantly influence the next step of cell growth or reproduction as cell cycle arrest by integrating environmental and internal signals.

In *C. albicans*, the carbon source sensing protein *Ca*Gpa2 not only regulated the cAMP level, but it also inhibited pheromone-mediated cell cycle arrest; the absence of *Ca*Gpa2 caused pheromone hypersensitivity and mating (Dignard et al., 2008). *Ca*Gpa2 was also important for activating the mating MAPK pathway, which showed a link between the nutrient-sensing pathway and the pheromone-responsive pathway (Bennett and Johnson, 2010). The *A. nidulans An*GprD protein regulates hyphal growth and conidial germination and represses sexual development during growth on glucose. In *A. fumigatus*, although GrpC and GprD are similar to the *S. cerevisiae* glucose receptor Gpr1, experiments showed that they were not involved in glucose and cAMP sensing. *Afu*GprC and *Afu*GprD proteins regulated growth, morphogenesis, reactive oxygen species (ROS), and temperature tolerance, and virulence in a murine model of pulmonary aspergillosis, although it had an opposing influence on the transcriptional regulation of primary and secondary metabolism (Gehrke et al., 2010). In *A. flavus*, inactivating each of 15 GPCR proteins separately showed mixed effects on the response to carbon sources, nitrogen sources, lipid molecules, environment stress signals, cell growth, conidiation, production of secondary metabolites, and virulence; two or more these responses were altered in several null mutants (Cabrera et al., 2015). It seems that GPCR-mediated signal transduction pathways cross each other in downstream cascades, which resulted in different outcomes? that were expected for a single GPCR target (Martín et al., 2019). Therefore, signals generated by distinct GPCR-mediated nutrient and pheromone-sensing pathways are potentially integrated into one biological outcome through downstream, dual-function signaling elements. In addition, GPCRs are adapted to detect multiple environmental cues and to bind multiple ligands to induce different signaling pathways. Thus, the interlinked signaling pathways are modulated differentially and fine-tuned to regulate multiple aspects of fungal development, metabolism, and virulence.

## Potential Role of GPCRs in a Fungal-Sensing Environment

The secondary metabolism biosynthetic genes in the fungal genome are clustered on the chromosome, which contain several key structural genes that encode multimodular enzymes that belong to the polyketide synthases (PKSs) or non-ribosomal peptide synthetases (NRPSs) families. These enzymes facilitate the construction of the main scaffold structure of many secondary metabolites. Additional enzymes introduced various modifications to the original structure (Alkhayyat and Yu, 2014). This complex process requires one or more cluster-specific transcription factors (TFs) to regulate, which operates downstream of G-protein signaling. The regulation of global TFs related to environmental signals is also at an upper level of cluster-specific regulation.

**FIGURE 2:**
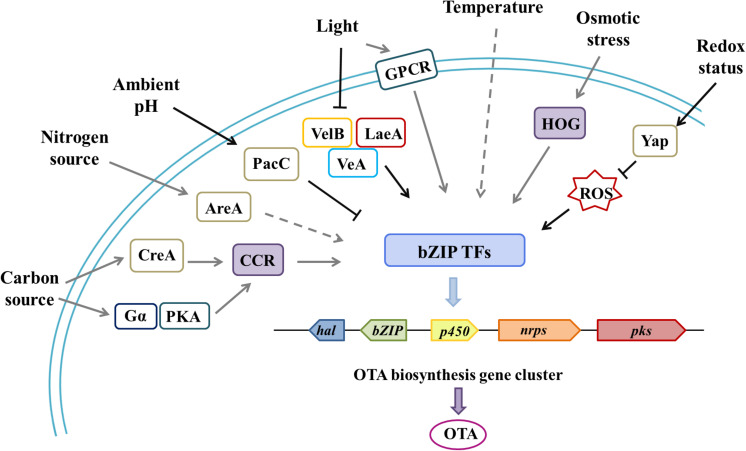
Environmental factors and associated regulatory elements that affect OTA biosynthesis through cluster-specific transcription factors.| Through various global transcription factors or specific pathways, related environmental cues affect the regulation of OTA gene clusters, which contain four highly conserved biosynthetic genes (*pks, nrps, hal/chl, p450*) and a cluster-specific, transcription factor gene (*bZIP*). bZIP plays a pivotal role in processing various environmental signals for a carbon source (CreA transcription factor and the carbon catabolite repression pathway), a nitrogen source (AreA transcription factor and the nitrogen catabolite repression pathway), light (Velvet complex proteins, VelB-VeA-LaeA, and the global regulator VosA), temperature and humidity, redox status (Yap transcription factor and ROS), and osmotic stress (the high osmolarity glycerol system). At the same time, there are also some examples where G protein pathways are partly involved in the perception of environmental cues, such as Gα(GanB) and PKA (PkaA) in *A. nidulans* and GPCR (NopA) in *A. fumigatus*. In this figure, the black solid arrows indicate connections verified in OTA-producing fungi; the gray solid arrows indicate connections that have been verified in other toxin-producting fungi, but not verified in OTA-producting fungi; and the gray dashed arrows indicate possible, yet unproven, connections.

For example, the most studied pathway-specific TF, AflR, which regulates the AF/sterigmatocystin (ST) biosynthetic gene cluster, was affected by CreA (carbon source), AreA (nitrogen source), Velvet (light), PacC (pH), and other global TFs (Macheleidt et al., 2016; Caceres et al., 2020; Gil-Serna et al., 2020). Other environmental factors, such as temperature, humidity, and osmotic pressure, also have a comprehensive influence on microbial behavior. High-similarity, specific gene clusters were found in several common OTA-produced *Aspergillus* and *Penicillium* species, which contained four highly conserved biosynthetic genes that encoded polyketide synthase (PKS), non-ribosomal peptide synthetase (NRPS), halogenate or chloroperoxidase (HAL/CHL), cytochrome P450 (P450), and a bZIP transcription factor (Antonia et al., 2016; Ferrara et al., 2016; Gil-Serna et al., 2018; Yan et al., 2018). bZIP transcription factor is supposed to be a specific regulator for secondary metabolite biosynthesis and the hub that accommodates various inputs that lead to a single output, and it altered the expression of all four biosynthetic genes (*pks, nrps, p450, hal*) (Figure 2; Reverberi et al., 2008; Nadia, 2015; Yan et al., 2018). Based on a similar mechanism of secondary metabolite production, we speculate that environmental signals may regulate OTA biosynthetic gene clusters in a cluster-specific, transcription factor-dependent manner; complex and integrated GPCRs likely play an important transfer role in this process.

### Carbon/Nitrogen Sources

Fungi can utilize a diverse array of nutrient sources, which include hexose, pentose, and complex saccharides. This nutritional plasticity may be reflected, therefore, in the dramatic expansion of putative GPCRs (Wang et al., 2020). A wider evaluation of the function of all 15 classical GPCRs in *A. flavus* revealed multiple GPCRs that were potentially involved in carbon sensing because the individual absence of the receptors GprA, GprC, GprJ, GprK, or GprR impaired growth on several carbon sources (Dierking and Pita, 2020). In addition to the GPCR mentioned above (Section “Perception of Nutrients”), nutrients also regulate life activities through global transcription factors. In the presence of favored carbon sources that can be metabolized rapidly to provide quick energy, fungi will repress the utilization of alternative, less preferred carbon sources, which is known as carbon catabolite repression (CCR) (Ruijter and Visser, 1997). The CCR mechanism is regulated in part by the C_2_H_2_ zinc-finger global transcription factor CreA, which represses the expression of genes required for catabolism of less preferable carbon sources, gluconeogenic genes, and nutrient acquisition genes in response to carbon starvation (Adnan et al., 2018). CreA up-regulated AF/ST biosynthesis by affecting the regulatory factor AflR (Hicks et al., 2001). Notably, loss of CreA in *A. nidulans*, *Fusarium oxysporum*, *P. chrysogenum*, and *Colletotrichum gloeosporoides* was fatal, but it did not affect the activity of *N. crassa*, *T. reesei*, and *A. fumigatus* (Ries et al., 2018). Recent studies revealed that Gα (GanB) and PKA (PkaA) participated in CreA-independent CCR, then regulated mycotoxin synthesis at the transcriptional level in *A. nidulans* (Kunitake et al., 2019; Figure 2). This study indicated that the G-protein signaling pathway and the global regulator pathway partially overlapped in their perception of a carbon source.

Nitrogen is another important nutrient that affects mycotoxin synthesis. In *A. carbonarius*, inorganic nitrogen and ammonium chloride strongly reduced the OTA level, but organic nitrogen promoted OTA yield (Tudzynski, 2014). Similarly, there was a nitrogen catabolite repression (NCR) for nitrogen sources, which was affected by the C_2_H_2_ zinc-finger, global transcription factor AreA (Wong et al., 2008). However, AreA contributed to, but was not essential for, virulence of *A. fumigatus* (Kmetzsch et al., 2011).

### Light

Photon is a type of ligand of rhodopsin-like GPCRs, which is a special animal neuronal photoreceptor that converts light signals into electrical signals. After photons are absorbed by rhodopsin, the voltage of the photoreceptor cell membrane is changed, thereby regulating life activities, and G protein plays a role in the conduction and amplification of this photoelectric signal (Nagy, 1991). In microorganisms, the bacterial rhodopsins bind retinal and act as light-driven proton pumps that pump protons from intracellular to extracellular regions? areas? to form a proton gradient, which is used by ATPase to synthesize ATP (Ignatov and Mosin, 2014). One class of fungal GPCR that is similar to bacterial rhodopsin has been identified. In *Neurospora crassa*, the NOP-1 receptor that is homologous to the bacterial opsin was verified and classified as a class IX GPCR. Results from Northern analysis supported light-based regulation of *nop-1* gene expression, and the NOP-1 protein functions as rhodopsin in *N. crassa* photobiology; (Bieszke et al., 1999) a unique homolog of NOP-1 was also identified in *Aspergillus* species (Supplementary Table 1).

The heterotrimeric Velvet complexes (VelB-VeA-LaeA) is a light-dependent regulator in fungi, which contain transcription factors (i.e., VelB, VelC, and VosA), and the global regulator (VeA) interacts with methyltransferase (LaeA) to regulate fungal development and secondary metabolism (Dasgupta et al., 2016; Zhang et al., 2018; Gil-Serna et al., 2020). VeA regulates the synthesis of ST in *A. nidulans* and AF in *A. flavus* (Eom et al., 2018); the Δ*veA* and Δ*velB* mutations impair ST production in *A. nidulans* (Kim et al., 2020) and OTA production in *A. carbonarius* (Crespo-Sempere et al., 2013) and *A. ochraceus* (Wang et al., 2019). The LaeA protein acts as a positive regulator on conidia production, OTA biosynthesis, and oxidative stress tolerance in *A. niger* (Ruitao et al., 2019). VeA is not a light sensor, but shows light-dependent mobility. Under light exposure, it occurs abundantly in the cytoplasm and is associated with filamentous bodies, but in the dark it is transferred into the nuclei by integrins. There, it is functionally active and interacts in concert with other proteins to form the velvet complex; it supports sexual reproduction and secondary metabolism in the dark and sporulation under light conditions (Calvo et al., 2016). Studies in *A. nidulans* linked the light-regulated Velvet pathway and GPCR, in which VeA regulators act as the bond. GprH, GprI (Class V), and GprM (Class VII) receptors collectively represent a carbon starvation-induced, nutrient sensing mechanism, which sensed glucose and propagated signals through the light-responsive VeA pathways and cAMP-PKA pathways to promote vegetative growth. GprH coordinated sexual development by regulating VeA nuclear localization and activity and then it repressed sexual development and ST production (Dos Reis et al., 2019).

Interestingly, different wavelengths are sensed by different light-receptors and have almost the opposite effect on mycotoxin production. This similar phenomenon was found in *Cherax quadricarinatus* because the activation of the Gq protein was related to the wavelength for light stimulation (Yan et al., 2003). Major OTA-produced *Aspergillus* and *Penicillium* spp. exposed to white light consistently reduced OTA production, whereas *Fusarium* spp. produced more fumonisin under light conditions (Fanelli et al., 2016). In *A. ochraceus*, *A. niger*, and *Penicillium* spp., red and blue wavelengths reduced OTA biosynthesis by modulating the level of expression of ochratoxin polyketide synthase. However, the red light for *A. carbonarius* and the yellow and green light for *A. steynii* caused increased OTA production (Fanelli et al., 2016). Different wavelengths of light are known to be accepted by different protein receptors, but the association with GPCRs needs to be explored further.

### Stress

Fungi have sophisticated signaling cascades to sense and to respond to different stressors, which include UV irradiation, temperature, osmotic shock, high salt, oxidative or nitrosative damage, and exposure to antifungal drugs (Arroyo et al., 2009). Mainly involved is the high osmolarity glycerol (HOG) system and its activated MAPK pathway. In addition to stress control, the fungal HOG pathway regulates cell-cycle progression, sexual development, and morphological differentiation. *Cryptoccus neoformans* has developed a specialized HOG pathway. Hog1 of *C. neoformans* is constitutively phosphorylated under normal conditions and represses sexual reproduction and the synthesis of capsule and melanin. In *P. verrucosum, P. nordicum*, and *A. carbonarius*, osmotic stress is associated with severe changes in OTA biosynthesis through the HOG pathway (De Assis et al., 2020). Metabolic reactions are triggered in part by cells in response to oxidative stress (redox state). In *A. parasiticus*, oxidative stress represented by intracellular ROS accumulation, enhanced AF production, but the application of antioxidants, such as butanol, reduced AF production (Jayashree and Subramanyam, 2000; Reverberi et al., 2005). The transcription factor Yap1 in *S. cerevisiae* controlled toxins produced in response to oxidative stress. Deletion of the *Yap-1* homologous gene *Aoyap1* in *A. ochraceus* damaged the activity of superoxide dismutases and catalases and, therefore, did not effectively scavenge high-level ROS to maintain the redox balance, which triggered the biosynthesis of OTA (Reverberi et al., 2012). Yap1 orthologs are coregulators of oxidative stress response with secondary metabolism in other aspergilli, such as *napA* in *A. nidulans*, *Apyap1* in *A. parasiticus*, and *Afap1* in *A. flavus* (Reverberi et al., 2007, 2008, 2012; Yin et al., 2013; Guan et al., 2019; Mendoza-Martinez et al., 2020). On the other hand, ROS catalyzed the production of oxylipin non-enzymatically, which is the ligand of receptors GprC and GprD, and they affected cellular activity that included toxin synthesis (described below). However, evidence for a strong link between the redox state and GPCR is still lacking.

### pH

G-protein-coupled receptors are mainly located on the surface of cell membranes and are regularly exposed to a wide range of dynamic pH microenvironments. Human OGR1 and GPR4 are typical proton-sensing, G-protein coupled receptors involved in pH homeostasis. GPR4, GPR65, and GPR68 receptors can be activated directly by pH and elicit cAMP formation (Rowe et al., 2020). However, no fungal proton-sensing GPCRs have been reported. In fungi, the perception of pH is closely related to the PacC pH adaptation signaling pathway. The dedicated proteins PalH, PalI, PalF, PalC, PalA, and PalB transmit environmental pH changes to transcription factor PacC (Cornet and Gaillardin, 2014). PacC effectively inhibits the expression of the known acid-expressed genes and undergoes pH-dependent proteolytic cleavage under alkaline ambient pH conditions, and then it regulates neutral-alkaline sensing in filamentous fungi (Tilburn et al., 1995; Penalva et al., 2008; Bignell, 2012). PacC is involved in the synthesis of secondary metabolites in fungi that activates penicillin biosynthesis at an alkaline pH (Brakhage, 1998), but it inhibits the biosynthesis of AF in *A. parasiticus*, ST in *A. nidulans*, OTA in *A. ochraceus*, fumonisin in *F. verticillioides* (Keller et al., 1997; O’callaghan et al., 2006), and gliotoxin in *A. fumigatus* (Kapetanakou et al., 2009). The regulation of pH in OTA synthesis is related to *pks* genes in the OTA biosynthesis gene cluster. Expression of the *otapksPN* gene in *P. nordicum* has been reported to be lower under acidic conditions below pH 5 (Geisen, 2004). Currently, there is no evidence that the pH sensor interacts with GPCRs in fungi. However, the structure of sensing external signals contained in pH sensors is similar to GPCR, which contains 7TMDs and a cytoplasmic C-terminus (Herranz et al., 2005; Hervas-Aguilar et al., 2010), and both regulate OTA synthesis at the transcriptional level. Therefore, we speculate that there is a certain connection between potential GPCRs and pH sensing in fungi, just like human pH-sensing GPCRs.

### Temperature and Humidity

Temperature and humidity both are key factors in the environmental regulation of fungal growth and secondary metabolites (MaríN et al., 1998; Tai et al., 2020; Wang et al., 2020). The minimum temperature for fungal growth increases with the enhanced inhibition of other environmental factors on growth, and mycotoxin production increases with the humidity increasing at the same temperature. However, there was not always a positive correlation between water activity and mycotoxin production in different cultures. The temperature range for mycotoxin production is stricter than for fungal growth. For example, suboptimal growth conditions that enhanced mycotoxin production is the optimum temperature for the biosynthesis of AF (i.e., 30°C), but the optimum temperature for *Aspergillus* growth is 37°C (Marc et al., 2002). The RNA-seq results of Yu et al. (year?) showed that most of the genes involved in AF synthesis, which included the cluster-specific regulatory genes *aflR* and *aflS*, were highly upregulated at low temperatures (Yu et al., 2011). Affeldt et al. (year?) annotated 15 GPCR genes in the *A. flavus* genome. The expression of *gprC*, *gprD*, *gprF*, *gprG*, and *gprO* were significantly different at three temperatures (20°C, 28°C, and 37°C). The deletion of either *gprC* or *gprD* resulted in a severe, temperature-dependent growth defect that was independent of the carbon source in *A. fumigatus* (Gehrke et al., 2010; Han et al., 2019). Knockout of *gprD* at a low temperature (20°C) increased the yield of AF, but there was no significant difference between wild-type and mutant under 28°C and 37°C (Han et al., 2019); this may have been due to the existence of alternative pathways to maintain normal biological functions.

The type of GPCR receptor and their ligands are being explored continuously, and the crossover phenomenon suggests that we cannot completely separate the G-protein signaling pathway and environmental factors perception for the regulation of OTA synthesis. Macroscopically, the dominant influence of temperature and humidity on OTA yield has been observed, and oxidative stress also plays a significant role; common stimuli, such as light and pH, also play a role. Indeed, studies have linked this regulation with GPCR. Although a large number of environmental factors influence the phenotypic phenomena associated with OTA biosynthesis, further confirmation of these is lacking. GPCRs play a critical role in perceiving these environmental stimuli and regulating the appropriate signaling pathways. However, the importance of GPCRs and the functional connections in regulating secondary metabolism are unknown.

## GPCRs Mediate Trans-Kingdom Communication

The natural interactions of fungi with bacteria, and cross-talks between infecting fungi and their infected hosts, induce many response signals that are sensed by fungi. Fungal G-protein signaling pathways that regulate cell behavior are also influenced by signaling molecules derived within and between fungal species (Fischer and Keller, 2016; Van Dijck et al., 2017), which implies that communication between species is partly mediated through GPCRs. This suggests that further study of these communication events is necessary to find targets to control mycotoxin biosynthesis.

### Fungal Quorum Sensing

Fungal QS is one of the main mechanisms for intra- and interspecific communication. Hormone-like small molecular compounds known as quorum-sensing molecules (QSMs) are secreted by fungi and enter into other cells through specific transporters and activate the expression of corresponding genes in a density-dependent manner within cells?. The QS mechanism plays a role in monitoring population density and regulating the physiology of fungal cells, that affect growth, sexual/asexual reproduction, apoptosis, secondary metabolism, and pathogenesis. This mechanism causes fungi to coordinate their actions and to enhance their survival, host immune evasion, and infection ability to adapt to environmental changes. Currently, verified fungal QSMs include alcohols, oxylipins, small molecule peptide pheromones, and certain volatile substances (Wongsuk et al., 2016; Barriuso et al., 2018; Padder et al., 2018). The perception of pheromones and their effects on fungal biology are mediated by GPCRs, and oxylipin is also a class of ligand for GPCRs.

Oxylipins, which is a large family of enzymatic or non-enzymatic oxidation products of fatty acids, are common signaling molecules in animals, plants, and microorganisms (Wasternack and Feussner, 2018). Treatment of *A. nidulans* wild-type with exogenous oxylipins resulted in cAMP accumulation, but this could be prevented in the absence of the *gprD* gene (De et al., 2013). Indeed, studies on both the GprD protein of *A. nidulans* and GprC and/or GprD proteins of *A. fumigatus* revealed that oxylipins were likely to be a type of ligand of these fungal GPCRs and to activate the cAMP pathway (Affeldt et al., 2012).

9S-Hydroxyoctadecadienoic acid (9-HODE), 13S-Hydroxyoctadecadienoic acid (13-HODE), and their derivatives that are derived from linoleic acid, act as crucial signals and elicitors of secondary metabolites in fungi and plants. 9(S)-HODE inhibited *A. ochraceus* sporulation and promoted the production of OTA, but 13(S)-HODE promoted the sporulation and inhibited the production of OTA (Reverberi et al., 2010). 13(S)-HPODE inhibited the expression of mycotoxin synthesis genes of *A. parasiticus* (*ver-1*) and *A. nidulans* (*stcU*) and significantly reduced the production of AFB1 and ST, although the inhibition effect at the same concentration of 9(S)-HPODE was not obvious (Burow et al., 1997).

Precocious sexual inducer (PSI) factors are mixed oxylipin signals produced by PpoA-C oxygenases, which are the main oxidases that regulate oxylipin synthesis in *Aspergillus* species, and the ratio of psiA-C determines if fungi enter sexual or asexual development (Fischer and Keller, 2016). *A. fumigatus* and *A. flavus* can produce the secreted PpoA oxylipin 5,8-dihydroxyoctadecadienoic acid (5,8-diHODE), which use a model of an autocrine-like mechanism to regulate hyphal branching. Also, the rice blast pathogen *Magnaporthe grisea* produces the branching-inducing oxylipin 7,8-diHODE. When exposed to exogenous 5,8-diHODE, *M. grisea* germlings differentiated predominantly into appressoria, the infectious structure required for plant penetration (Niu et al., 2020). On the other hand, *M. grisea* can produce the autocrine signal 7,8-diHODE to induce branching, and it is proposed that there is a cross-genera recognition of fungal dihydroxyl oxylipins between *Aspergillus* and *M. grisea*, where the 5,8-diHODE acts as a paracrine signaling molecule between cells of some fungal species.

Knocking out the *ppo* genes in *A. nidulans* reduced the production of oxylipins and then it disrupted the balance of sexual/asexual sporulation, although the double Δ*ppoA*Δ*ppoC* mutants and triple Δ*ppoA–C* mutants lost their ability to produce the mycotoxin ST, but showed an overproduction of the antibiotic penicillin. It also weakened the fungal ability to colonize in peanuts and maize hosts (Tsitsigiannis and Keller, 2010). These phenotypes were similar to the constitutively activated Gα, *An*Fad^*AG*42*R*^, which suppressed the ST inducer gene *AnAflR*, but enhanced the penicillin biosynthetic gene *AnIpnA* that was mediated through the cAMP-PKA pathway (Tag et al., 2010). Disruption of *Ppo* orthologs also reduced T2 production in *Fusarium sporotrichioides* (Mcdonald et al., 2003). After knocking out all five dioxygenase genes (*ppoA-D* and *lox*), *A. flavus* lost its density-dependent regulation of sporulation and AF production; both the Δ*ppoC* and Δ*lox* mutant strains produced high levels of AF at any population density (Brown et al., 2009). Oxylipins added at different concentrations exogenously affected the normal morphological development of AF. In addition, when fungi were exposed to oxidative stress, such as an increased ROS level, this led to the synthesis of oxylipins and induced toxin production. Therefore, the QS mechanism represented by fungal oxylipins can impact fungal sporulation, mycotoxin production, and virulence in a density-dependent regulation that is partly mediated by GPCRs.

### Interspecies Fungal Communication

Fungi communicate with other fungi and bacteria to produce signaling molecules that also regulate their growth form and virulence through G-protein signaling pathways. For example, in co-cultivation of *C. albicans* and *A. nidulans*, farnesol produced by the former affected the latter by inhibiting its growth and/or inducing apoptosis (Semighini et al., 2006). Exposure of *A. nidulans* to farnesol did not influence the emergence of the germ-tube, but relied on G-protein signaling to affect mitochondrial function and ROS production; what was dependent? dependent on autophagy and PKC signaling, which caused cell apoptosis. Experiments have shown that the *ΔflbA* mutation affected activity of the Gα protein FadA that caused a significant increase in the sensitivity of farnesol, which revealed the signal transduction role of the FadA-G protein complex in this fungal interspecies communication process (Semighini, 2006; Savoldi et al., 2010). Conversely, another QSM pantothenic acid has positive impacts on fungal growth. Both *C. neoformans*-conditioned media, which contained *C. neoformans* autocrine pantothenic acid, and exogenous pantothenic acid increased the growth of *C. albicans* and *S. cerevisiae* (Albuquerque et al., 2014).

Fungi and bacteria can interact through signaling molecules. *Pseudomonas aeruginosa* is often found with *C. albicans* in mixed mammalian infections; the former can grow on and damage filamentous hyphae, but not budding yeast cells, and the bacterial QSM homoseryl lactone inhibits filamentation (Hogan et al., 2004; Cugini et al., 2010). Recent studies have shown that in mixed *C. albicans* and *S. aureus* biofilm, farnesol produced by the former induced ROS in the latter bacterium, which resulted in the up-regulation of drug efflux pumps that protected bacterial cells from antibiotic damage (Kong et al., 2017).

### Host–Pathogen Communication

**FIGURE 3:**
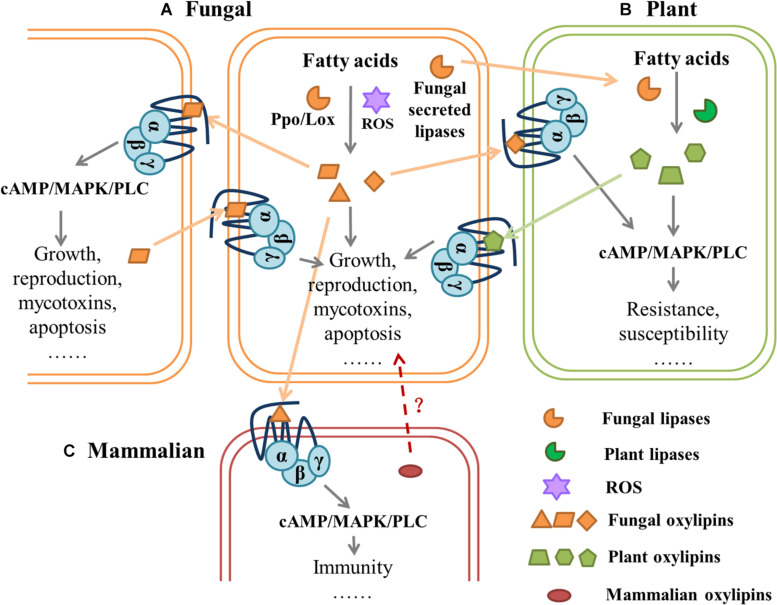
Hypothetical model of mediated communication of G-protein-coupled receptors between fungi and their hosts.| **(A)** Fungal lipoxygenase (Lox) and psi-producing oxygenases (Ppo) or reactive oxygen species (ROS) can catalyze the oxidation of fatty acids (FAs) to oxylipins, which affect fungal behavior through downstream pathways. Different species of fungi can sense oxylipins secreted *in vitro* by GPCRs, which regulate cell growth, reproduction, mycotoxins, and apoptosis. **(B)** Fungal lipases are secreted into plant cells where fatty acid substrates are cleaved and processed by fungi- secreted lipoxygenases and/or plant lipoxygenases for oxylipin production. Plant-produced oxylipins are perceived and exploited by fungi to regulate GPCR-mediated behavior. **(C)** In mammalian cells, different signals from microbial-derived oxylipids, mediate inflammatory responses, and fungi can affect host physiology through GPCRs. It is also possible that mammalian oxylipins affect fungal activity. All fungal lipases and oxylipins are orange, all plant lipases and oxylipins are green, mammalian oxylipins are red. Dashed arrows with the question mark (?) indicates hypothetical interactions, and solid arrows represent proven interactions.

There is a complex GPCR-mediated communication between fungi and their hosts, in which oxylipins may play a completely different, but crucial role (Figure 3). For example, Gpr1 of *S. cerevisiae* showed structural and functional homology with mammalian GPCRs, and 9(S)-HODE and other oxidized free fatty acids bound to the mammalian G2A (oxylipins receptor), which inhibited cellular cAMP accumulation and MAP kinase activation (Obinata et al., 2005). Further, different eicosanoids mediated inflammatory responses through GPCRs in mammals (Dennis and Norris, 2015). *Ca*Gpr1 in *C. albicans* detected l-lactate released by the gut microorganism *Lactobacillus reuteri*, which promoted fungal β-glucan masking and evaded the mammalian immune system (Ballou et al., 2016). *C. albicans* can secrete QSMs tyrosol and farnesol; the former hindered the killing of mammalian host neutrophils by inhibiting ROS production (Josef et al., 2010), and the latter induced macrophage apoptosis (Abe et al., 2010). In the entomopathogenic *Metarhizium* species, deletion of the GPCR *Mr*Gpr8 (Class XIV Pth11-like GPCR) substantially impaired the nucleus translocation in MAPK, which resulted in the failure of appressorium to form on different substrates and the loss of virulence during topical infection of insects. The *ΔMrGpr8* mutants could not be rescued with the addition of cAMP for appressorium formation (Shang et al., 2020). This model recognizes that the G protein signaling pathway can integrate intra- and interspecific signal transmission and cellular activity. In humans and mice, it is well-established that GPCRs detect microbial-derived signals and that the microbiota can affect host physiology through GPCRs (Dierking and Pita, 2020).

Plant hosts secrete a variety of elicitors that induce defense responses against the infecting fungi, but fungal signal molecules also modulate host immunity and damage host cells (Ariyo et al., 1997; Liu et al., 2001; Benz et al., 2014). The plant oxylipin jasmonate inhibited fungal reproduction and secondary metabolism as a defense against necrotrophic fungal pathogens (Calvo et al., 1999), and it can also promote *F. oxysporum* infection (Thatcher et al., 2009). Plant oxylipins seem to have a completely different effect on fungi. Linoleic acid and 9S-HPODE promoted mycotoxin synthesis in *Aspergillus*, whereas 13S-HPODE inhibited it (Burow et al., 1997). *A. ochraceus* prefers to infect crops that contain more fatty acid and produce more OTA (Caiyan et al., 2017), which is speculated to be related to the oxidation of plant fatty acids to produce oxylipins. Who proposed? proposed a hypothetical model that the host-derived oxylipins can bind with fungal GPCR to regulate growth, sporulation, and synthesis of mycotoxin; on the other hand, these oxylipins may stimulate biosynthesis of fungal oxylipins. The psiB factor in *Aspergillus* is also derived from linoleic acid.

The complementation of Δ*ppoA*Δ*ppoC* mutants with the maize *ZmLOX3* gene restored conidiation and, thus, the ability of plant oxylipins to mimic or to interfere with fungal signaling may be due to structural similarities (Brodhagen et al., 2010). Destroying the *ZmLOX3* gene of maize caused a deficiency in 9-LOX derivatives, which compromised conidiation, pathogenicity, and mycotoxin production of maize pathogenic *Fusarium verticillioides*, and this promoted resistance to other fungal pathogens (Wilson et al., 2001). On the contrary, maize that lacked the *lox3* gene were more susceptible to *Aspergillus* infection, and AF contamination was more serious, which indicated that host oxylipins promoted pathogenesis in addition to resisting pathogenic (Reverberi et al., 2010; Yan et al., 2015).

Another mechanism proposed in *F. graminearum* is that *Aspergillus* can secrete lipases and LOX in host cells to regulate host lipid metabolism by cleaving off free fatty acids and oxidizing them to produce oxylipins. Fungi can then use host-derived oxylipins to facilitate invasive growth, spores, and mycotoxin production (Voigt et al., 2010). Similarly, host-derived 3-hydroxyoxylipin promoted the ability of *C. albicans* to grow and become more virulent within mammalian cells, whereas salicylic acid treatment inhibited fungal development and biofilm formation (Tsitsigiannis and Keller, 2007).

In summary, QS plays an important role in intra- and interspecies communication, which impact fungal development, mycotoxin regulation, and disease. The key role of oxylipins and their status as GPCR ligands suggest that these mechanisms are related to the G-protein signaling pathway, but the receptors capable of sensing these QSMs and the specific mechanisms remain to be discovered.

## Conclusion

Biological detoxification methods have greater safety, availability, and cost-effectiveness than physical and chemical detoxification methods. Currently, research advances in OTA biodetoxification has been made in degradation, adsorption, or enzymatic degradation[9]. With the development of bioinformatics and molecular biology, researchers have turned to studies of molecular biological mechanisms to crack the code of fungal physiology. Fungal GPCRs have been proposed as targets for controlling mycotoxins. The importance of the GPCR signaling pathway to fungal biology and virulence is underexplored, and only a limited number of receptors have been shown to regulate OTA synthesis directly. However, there are a large number of unclassified orphan GPCRs. The ligands of these orphan GPCRs are unidentified, and their physiological role is yet to be determined, which implies a huge number of possible targets link the perception of extracellular signals with mycotoxin synthesis, such as pH-sensing GPCRs. Another area worthy of further study is that of quorum sensing inhibition through the blockage of signal production (i.e., Quorum Quenching), which limits fungal growth and mycotoxin production by affecting intercellular communication. This can be achieved partly by inhibiting the GPCR’s reception of QSMs such as oxylipins (Turan and Engin, 2018). Therefore, fungal-specific GPCRs represent promising and unexplored targets to potentially intervene or to reduce the impact of mycotoxin contamination and fungal diseases. Deeper understanding of fungal GPCRs will enhance our ability to develop novel strategies in agricultural and clinical settings to promote human, animal, plant, and even ecosystem health.

## Author Contributions

JG contributed to conception and design of the study, and wrote the first draft of the manuscript. XX wrote sections of the manuscript. KH final approval of the version to be published. ZL wrote sections of the manuscript and final approval of the version to be published. All authors contributed to manuscript revision, read, and approved the submitted version.

## Conflict of Interest

The authors declare that the research was conducted in the absence of any commercial or financial relationships that could be construed as a potential conflict of interest.

## Supplementary Material

Supplementary Table 1 Classification of G-protein-coupled receptors in fungi (Martín et al., 2019).
