# Supplementary material for: Fungal G-Protein-Coupled Receptors: A Promising Mediator of the Impact of Extracellular Signals on Biosynthesis of Ochratoxin A
Source: Front Microbiol. 2021 Feb 12;12:631392. doi: 10.3389/fmicb.2021.631392 (PMC7907439; doi:10.3389/fmicb.2021.631392)
Supplement: Supplementary Table 1 — Classification of G-protein-coupled receptors in fungi (Martín et al., 2019). [file Table_1.DOCX]

| **GPCR** | **Class** | **Biological function** | **Species** | **Ligands** | **Regulating behaviors** | **References** |
| --- | --- | --- | --- | --- | --- | --- |
| Classical | I | α-factor pheromone receptor | *A. fumigatus*  (GprA) | / | sexual/ asexual development | ^[35]^ |
|  |  |  | *A. nidulans*  (GprA) | / | self-fertilization,  sexual development | ^[41]^ |
|  |  |  | *A. oryzae*  (GprA) | / | sexual/ asexual development | ^[32]^ |
|  |  |  | *Saccharomyces cerevisiae*  (Ste2) | α-factor | cell division and conjugation. | ^[42]^ |
|  |  |  | *N. crassa*  (PRE-2) | pheromone CCG-4 | sexual/asexual development  (post-fertilization) | ^[43]^ |
|  | II | a-factor pheromone receptor | *A. fumigatus*  (GprB) | / | sexual/ asexual development | ^[35]^ |
|  |  |  | *A. nidulans*  (GprB) | / | self-fertilization,  sexual development | ^[41]^ |
|  |  |  | *A. oryzae*  (GprB) | / | sexual/ asexual development | ^[32]^ |
|  |  |  | *S.cerevisiae*  (Ste3, Ste6) | a-factor | sexual/ asexual development, multiple drug resistance | ^[42]^ |
|  |  |  | *N. crassa*  (PRE-1) | GNA-1 | sexual development | ^[44]^ |
|  | Ⅲ | Carbon source sensors | *A. fumigatus*  (GprC, GprD) | carbohydrate,  oxylipin,  cAMP | mycotoxin production  (fumitremorgin, pseurotin A）,  adaptation of stress signaling | ^[45]^ |
|  |  |  | *A. flavus*  (GprC, GprD) | carbohydrate,  oxylipin,  cAMP | quorum sensing, sporulation, sclerotia formation, and AF biosynthesis | ^[46]^ |
|  |  |  | *A. oryzae*  (GprC, GprD) | carbohydrate | DHN-melanin production | ^[32]^ |
|  |  |  | *S.cerevisiae* (Gpr1) | glucose sucrose | pseudohyphal differentiation | ^[47]^ |
|  |  |  | *N.crassa*  (GPR-4) | glycerol | asexual development | ^[48]^ |
|  |  |  | *A. nidulans*  (GprC, GprD, GprE) | carbohydrate | sterigmatocystin production, hyphal growth and conidial germination | ^[44]^ |
|  | IV | Nitrogen/Nutrient source sensors | *A. fumigatus*  (GprF, GprG, GprJ) | nitrogen source | DHN-melanin production | ^[35]^ |
|  |  |  | *A. oryzae*  (GprF, GprG, GprJ) | nitrogen source | / | ^[32]^ |
|  |  |  | *Cryptococcus neoforme*(Gpr4) | methionine | capsule production,  asexual development | ^[17]^ |
|  |  |  | *S. cerevisiae* (Ypq1p) | amino acids | nutrition | ^[49]^ |
|  |  |  | *N. crassa*  (Gpr-5,Gpr-6) | amino acids | nutrition | ^[50]^ |
|  |  |  | *A. nidulans*  (GprF, GprG, GprJ) | nitrogen source | / | ^[44]^ |
|  | V | cAMP receptor-like (CRL) | *A. fumigatus*  (GprH, GprI, GprL) | carbon,  amino acid | hyphal growth | ^[35]^ |
|  |  |  | *A. nidulans*  (GprH, GprI) | glucose,  tryptophan,  cAMP | Primary metabolism, hyphal growth and sexual reproduction | ^[38]^ |
|  |  |  | *A. oryzae*  (GprH) | / | / | ^[32]^ |
|  |  |  | *Dictyostelium discoideum*  (CrlA) | cAMP | cell growth and developmental morphogenesis | ^[51]^ |
|  |  |  | *N. crassa*  (Gpr-1、2、3) | cAMP | sexual/ asexual development | ^[50]^ |
|  |  |  | *Trichoderma atroviride*  (Gpr-1-4) | carbohydrate ,  cAMP | conidial germination,  vegetative growth | ^[52]^ |
|  | IX | Microbial opsins | *A. fumigatus*  (NopA) | light | secondary metabolism | ^[35]^ |
|  |  |  | *A. nidulans*  （NopA） | / | / | ^[32]^ |
|  |  |  | *A. oryzae*  （NopA） | / | / | ^[32]^ |
|  |  |  | *N. crassa*  (NOP-1, ORP-1) | light | asexual developmentact | ^[32]^ |
| Novel | VI | RGS domain- containing | *A. fumigatus*  (GprK) | carbon sources | asexual development, gliotoxin production, oxidative stress response | ^[53]^ |
|  |  |  | *A. nidulans*  (GprK) | / | / | ^[32]^ |
|  |  |  | *A. oryzae*  (GprK) | / | / | ^[32]^ |
|  | VII | Homelogous to rat growth hormone releasing factor receptors | *A. fumigatus*  (GprM) | xylose | DHN-melanin production | ^[54]^ |
|  |  |  | *A. nidulans*  (GprM, GprN) | / | / | ^[32]^ |
|  |  |  | *A. oryzae*  (GprM) |  |  | ^[32]^ |
|  | VⅢ | mPR-like/PAQR (homologous to yeast Izh, zinc regulators) | *A. fumigatus*  (GprO、GprP) | steroid | metabolism of lipids and phosphate | ^[32]^ |
|  |  |  | *A. nidulans*  (GprO, GprP) | / | / | ^[32]^ |
|  |  |  | *A. oryzae*  (GprO, GprP) | / | / | ^[32]^ |
|  |  |  | *S. cereviside*  (IZH2、 IZH3) | progestin and progesterone | drug resistance (polyene antifungal), fungal filamentation | ^[55]^ |
|  | X | Lung 7TM Superfamily or PTMI-like GPCR | *S. cereviside*  (Ptm1) | / | / | ^[56]^ |
|  | XI | GPCR89/ABA-GPCR | *N crassa*  (Gpr-12) | / | / | ^[35]^ |
|  |  |  | *S. cereviside*  (HGI1) | glucose | high glucose stress response | ^[57]^ |
|  | XII | Family C-like | *N. crassa*  (Gpr-13) | / | / | ^[35]^ |
|  | XⅢ | DUF300 superfamily/PSGPR11 | *N. crassa*  (Gpr-14) | / | / | ^[35]^ |
| Structurally different | XIV | Pth11 and Pthl1-like | *N. crassa*  (Gpr-15, Gpr-23, Gpr-29) | cellulose materials,  plant cell walls | sexual/ asexual development,  hyphal growth | ^[50]^ |

[35]Grice, C.M., Bertuzzi, M., Bignell, E.M., 2013. Receptor-mediated signalling in Aspergillus fumigatus. Front. Microbiol. 4, 26.

[41]Seo J A , Han K H , Yu J H . The gprA and gprB genes encode putative G protein-coupled receptors required for self-fertilization in Aspergillus nidulans[J]. Molecular Microbiology, 2004.

[42]Kim J , Bortz E , Zhong H , et al. Localization and signaling of G(beta) subunit Ste4p are controlled by a-factor receptor and the a-specific protein Asg7p.[J]. Molecular & Cellular Biology, 2000, 20(23):8826.

[43]Kim H , Wright S J , Park G , et al. Roles for Receptors, Pheromones, G Proteins, and Mating Type Genes During Sexual Reproduction in Neurospora crassa[J]. Genetics, 2012, 190(4):1389-404.

[44]Hyojeong, Kim, Katherine, et al. A pheromone receptor gene, pre-1, is essential for mating type-specific directional growth and fusion of trichogynes and female fertility in Neurospora crassa[J]. Molecular Microbiology, 2004, 52(6):1781-1798.

[45]Gehrke A , Heinekamp T , Jacobsen I D , et al. Heptahelical receptors GprC and GprD of Aspergillus fumigatus Are essential regulators of colony growth, hyphal morphogenesis, and virulence.[J]. Applied and Environmental Microbiology, 2010, 76(12):3989-3998.

[46]Affeldt K J , Brodhagen M , Keller N P . Aspergillus Oxylipin Signaling and Quorum Sensing Pathways Depend on G Protein-Coupled Receptors[J]. Toxins, 2012, 4(9):695-717.

[47]Lorenz M C , Pan X . The G protein-coupled receptor Gpr1 is a nutrient sensor that regulates pseudohypal[J]. Genetics, 2000.

[48]Li L , Borkovich K A . GPR-4 Is a Predicted G-Protein-Coupled Receptor Required for Carbon Source-Dependent Asexual Growth and Development in Neurospora crassa[J]. Eukaryotic Cell, 2006, 5(8):1287-1300.

[44]Han, K.‐H., Seo, J.‐A. and Yu, J.‐H. (2004), A putative G protein‐coupled receptor negatively controls sexual development in Aspergillus nidulans. Molecular Microbiology, 51: 1333-1345.

[17]Xue C , Bahn Y S , Cox G M , et al. G protein-coupled receptor Gpr4 senses amino acids and activates the cAMP-PKA pathway in Cryptococcus neoformans.[J]. Molecular Biology of the Cell, 2006, 17(2):667.

[49]Sekito T , Nakamura K , Manabe K , et al. Loss of ATP-dependent lysine uptake in the vacuolar membrane vesicles of Saccharomyces cerevisiae ypq1 mutant[J]. Bioscience Biotechnology & Biochemistry, 2014, 78(7):1199-1202.

[50]Cabrera I E , Pacentine I V , Lim A , et al. Global Analysis of Predicted G Protein Coupled Receptor Genes in the Filamentous Fungus, Neurospora crassa[J]. G3: Genes|Genomes|Genetics, 2015, 5(12):2729-2743.

[38]Andrew N , Brown, Fernanda T , et al. G-protein coupled receptor-mediated nutrient sensing and developmental control in Aspergillus nidulans.[J]. Molecular microbiology, 2015.

[51]Raisley B , Zhang M , Hereld D , et al. A cAMP receptor-like G protein-coupled receptor with roles in growth regulation and development[J]. Developmental Biology, 2004, 265(2):433-445.

[52]Brunner K , Omann M , Pucher M E , et al. Trichoderma G protein-coupled receptors: functional characterisation of a cAMP receptor-like protein from Trichoderma atroviride[J]. Current Genetics, 2008, 54(6):283-299.

[32]Lafon A , Han K H , Seo J A , et al. G-protein and cAMP-mediated signaling in aspergilli: a genomic perspective[J]. Fungal Genetics & Biology, 2006, 43(7):490-502.

[53]Jung M G , Kim S S , Yu J H , et al. Characterization of gprK Encoding a Putative Hybrid G-Protein-Coupled Receptor in Aspergillus fumigatus[J]. PLoS ONE, 2016, 11(9):e0161312.

[54]FILHO A P D C, BRANCINI G T P, DE CASTRO P A, et al. Aspergillus fumigatus G-Protein Coupled Receptors GprM and GprJ Are Important for the Regulation of the Cell Wall Integrity Pathway, Secondary Metabolite Production, and Virulence [J]. mBio, 2020, 11(5):

[55]Villa N Y , Moussatche P , Chamberlin S G , et al. Phylogenetic and Preliminary Phenotypic Analysis of Yeast PAQR Receptors: Potential Antifungal Targets[J]. Journal of Molecular Evolution, 2011, 73(3-4):134-152.

[56]Inadome H , Noda Y , Adachi H , et al. Immunoisolaton of the Yeast Golgi Subcompartments and Characterization of a Novel Membrane Protein, Svp26, Discovered in the Sed5-Containing Compartments[J]. Molecular and Cellular Biology, 2005, 25(17):7696-7710.

[57]Gomar-Alba M , Jiménez-Martí, E, Del Olmo M . The Saccharomyces cerevisiae Hot1p regulated gene YHR087W (HGI1) has a role in translation upon high glucose concentration stress[J]. BMC Molecular Biology,13,1(2012-06-21), 2012, 13(1):19-19.
